# Ipsilateral Salpingopexy in a Case of Isolated Tubal Torsion

**DOI:** 10.7759/cureus.33326

**Published:** 2023-01-03

**Authors:** Shweta Mittal, Bhawani Shekhar

**Affiliations:** 1 Centre of IVF and Human Reproduction, Department of Obstetrics and Gynaecology, Sir Gangaram Hospital, Delhi, IND

**Keywords:** salpingopexy, detorsion, laparoscopy, prompt diagnosis, isolated tubal torsion

## Abstract

Isolated fallopian tube torsion (IFTT) is a rare emergency condition affecting young females. Due to the diagnostic dilemma, diagnosis of IFTT is often delayed leading to tubal necrosis and salpingectomy as the only choice of treatment. If diagnosed early, it can be managed conservatively by detorsion. Salpingopexy is an option described in the literature to prevent recurrence of this condition; however, evidence is scarce. This case report highlights the role of prompt diagnosis and management of IFTT. It describes a case of IFTT with paratubal cysts and a long tube in a young female, which was timely diagnosed and managed conservatively by laparoscopic detorsion, paratubal cystectomy, and ipsilateral salpingopexy.

## Introduction

Isolated fallopian tube torsion (IFTT) is an extremely rare condition with an incidence of one in 1.5 million, affecting young adolescents and young women of reproductive age [1]. It is a surgical emergency with challenging pre-operative diagnosis. In most cases, diagnosis is delayed, leading to tubal necrosis and salpingectomy as the only choice of treatment. If diagnosed early, it can be managed conservatively by detorsion. Salpingopexy is an option described in the literature to prevent recurrence of this condition as described in our case; however, evidence is scarce [2]. This case report highlights the role of prompt diagnosis and management of IFTT.

## Case presentation

A 24-year-old unmarried female with no past medical or surgical history presented to the emergency department in the morning with severe lower abdominal pain in the left iliac fossa since previous evening. Pain was acute in onset, not relieved with analgesics. The patient did not complain of any bowel or bladder symptoms. She was on day 12 of a 30-day menstrual cycle.

**Figure 1:**
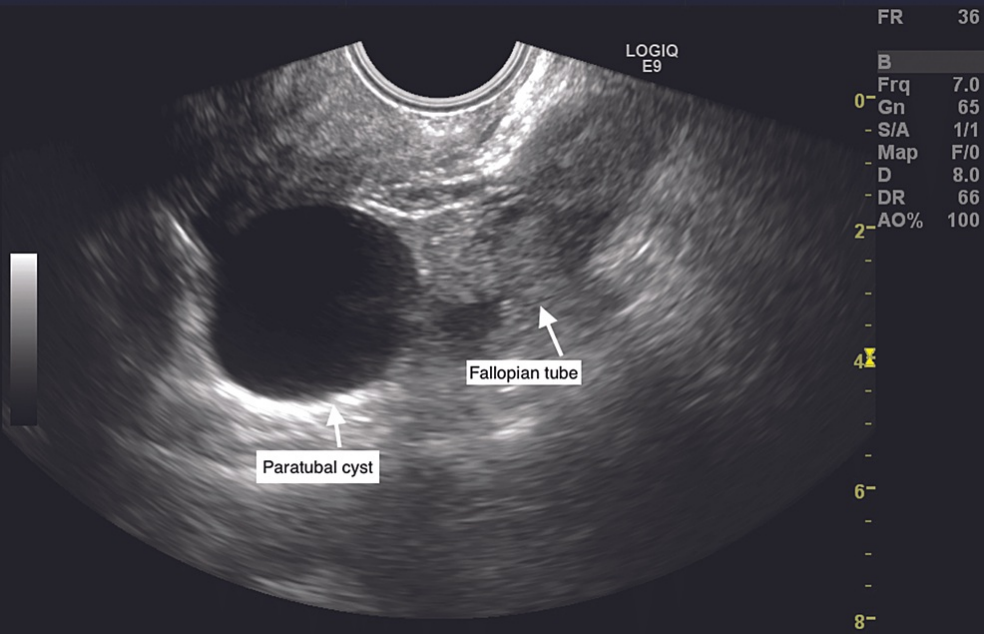
Left paratubal cyst and dilated left fallopian tube with echogenic contents

**Figure 2:**
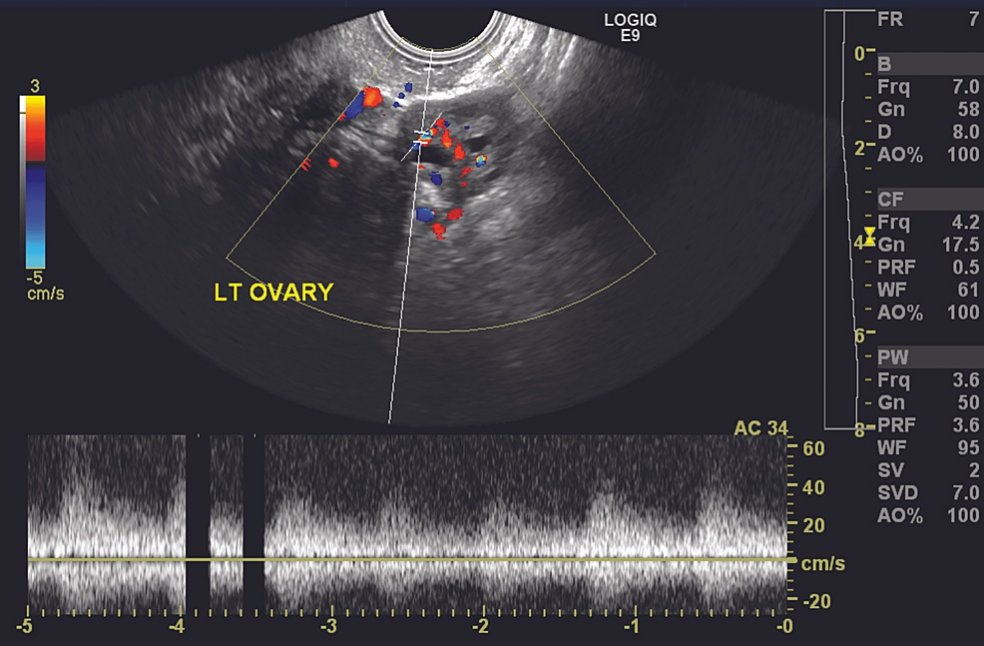
Transabdominal ultrasound revealing a normal appearing left ovary with normal blood flow on color Doppler

Clinical examination revealed tenderness in the left iliac fossa. On presentation, the patient was hemodynamically stable with a pulse rate of 96 beats/min and a blood pressure of 110/70 mm Hg. Urine pregnancy test was negative. Hemoglobin level was 12.4 g/dL, and total leucocyte count was 4,470/cumm. Liver kidney function tests and urine routine microscopy were within normal limits. Transabdominal ultrasound revealed normal size uterus with endometrial thickness of 6.4 mm. Clear cyst of size 3.6 x 3.3 cm was seen in the pouch of Douglas separate from the ovary. The left fallopian tube appeared mildly dilated with echogenic contents within it (Figure 1). The left ovary appeared normal. On color Doppler, blood flow to the left ovary was normal (Figure 2). The right ovary was normal. Minimal free fluid was seen in the pouch of Douglas. Bilateral kidneys were normal. Tubo-ovarian torsion seemed less likely as the left ovarian stroma appeared normal with normal blood flow to the left ovary on ultrasound. Isolated tubal torsion seemed the most likely diagnosis; however, considering rarity of this condition, tubo-ovarian torsion was not ruled out.

**Figure 3:**
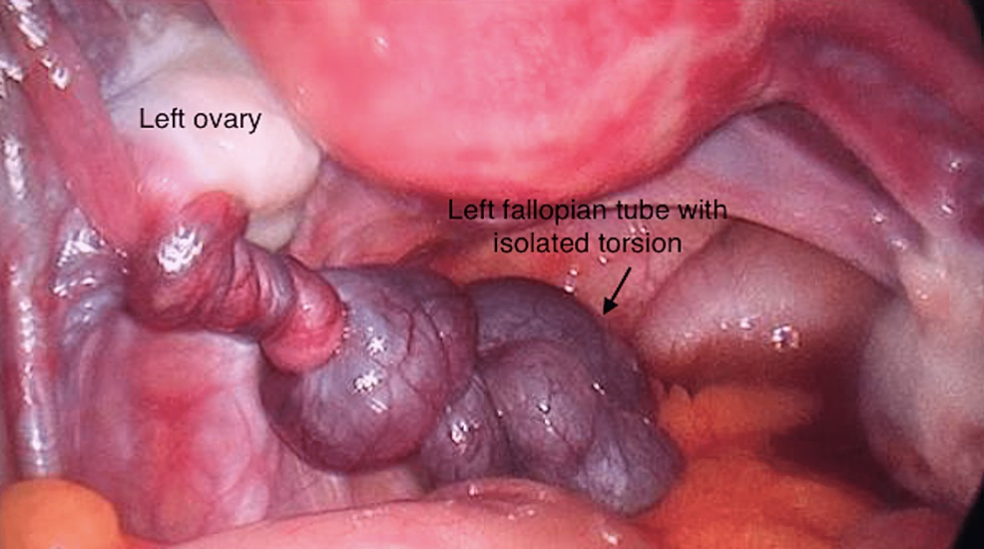
Left fallopian tube with isolated torsion

Intraoperative findings revealed bluish colored left fallopian tube with multiple twists on itself confirming fallopian tube torsion (Figure 3). Minimal free fluid was seen in the pouch of Douglas.

**Figure 4:**
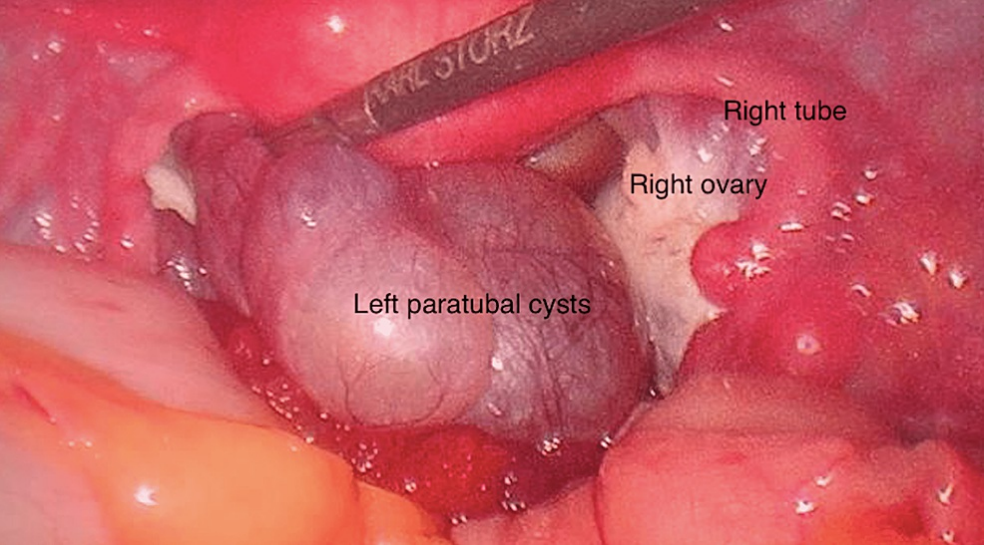
Left paratubal cysts in the pouch of Douglas

One 3-cm and two 1-cm paratubal cysts distinctly separate from the ovary were seen lying in the pouch of Douglas (Figure 4).

**Figure 5:**
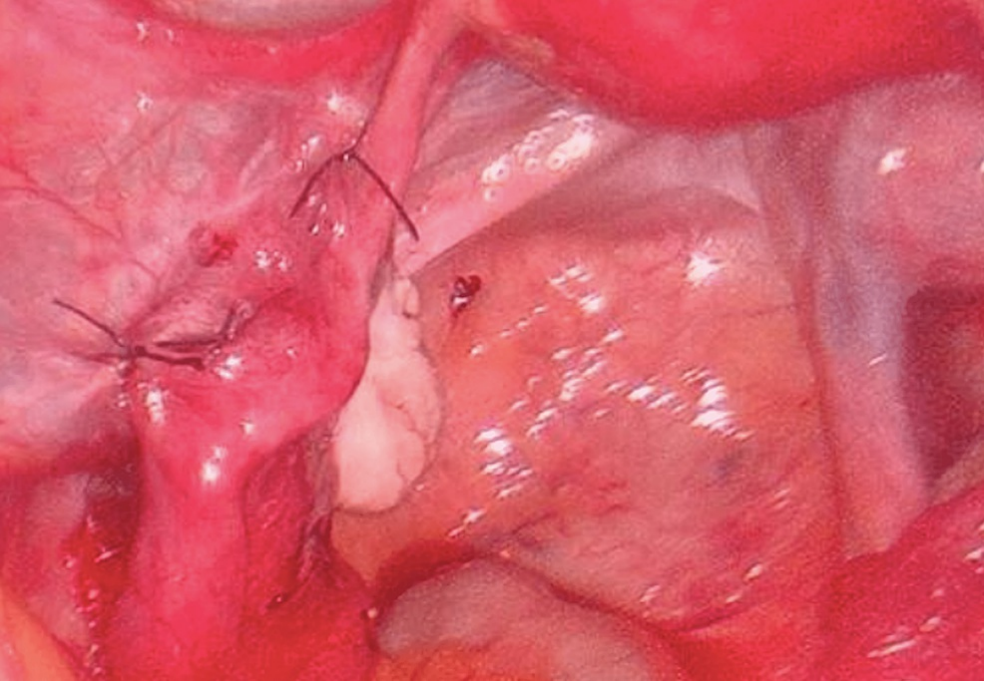
Left tube fixed at two sites: proximal part to the left ovarian ligament and distal part to the left infundibulopelvic ligament (salpingopexy)

The left ovary appeared normal with no evidence of torsion. No other abnormal findings were seen intra-abdominally. Left fallopian tube was detorted with atraumatic graspers and was found to have rotated seven times along the fallopian tube axis. On detorsion, the fallopian tube regained its natural color and was found to be longer than normal (one and a half times longer). Right fallopian tube was normal in length. Left paratubal cystectomy was performed. In view of the long length of the left fallopian tube and possibility of retorsion, decision to perform left salpingopexy was taken. Left fallopian tube was plicated at two sites using 2-0 vicryl suture: proximal tubal part to the left ovarian ligament and the mid tubal part to left mesosalpinx (Figure 5).

Hemostasis was ensured. Paratubal cysts were sent for histopathological examination. The patient had an uneventful postoperative course and was discharged on postoperative day 3. Histopathology revealed benign cysts. The patient followed up in the clinic till eight weeks postoperatively and had not experienced any further abdominal pain.

## Discussion

IFTT is torsion of the fallopian tube without concomitant ovarian torsion. Risk factors include intrinsic factors such as long or spiral tube, hydrosalpinx, pelvic inflammatory disease (PID), tubal ligation, or extrinsic factors such as adhesions, adnexal venous congestion, or paratubal cysts. The most common presentation is unilateral lower abdominal or flank pain with tenderness on palpation as was seen in our case. Patients may also experience other nonspecific symptoms such as nausea, vomiting, dysuria, or diarrhea [3]. It is suggested that IFTT predominantly appears on the right side, possibly because of partial immobilization of the left tube due to sigmoid mesentery [4]. It is also suggested that there is a greater tendency for patients with right-sided lower abdominal pain to be operated upon to exclude appendicitis [5]. However, in our case, the left side was involved. It is difficult to diagnose this entity on ultrasound due to highly variable findings. It is accurately diagnosed on ultrasound in 30% of the cases. Hence, the diagnosis of IFTT is mostly delayed [6]. Ultrasound findings that suggest IFTT include a thickened fallopian tube with internal debris or twisted tubal vasculature (whirlpool sign). Color Doppler may be useful in diagnosing IFTT, but normal tubal blood flow does not rule out the torsion [7]. IFTT should be considered in the differential diagnosis of acute lower abdominal pain, particularly if an adnexal cyst is seen on ultrasound. Even if ultrasound is inconclusive, surgery should not be delayed for further imaging studies. In our case, ultrasound was able to diagnose the condition accurately, which aided in prompt management of the case. Diagnosis was confirmed by an urgent laparoscopy. Due to timely diagnosis and intervention, removal of the tube (salpingectomy) could be avoided as the tube had not undergone necrosis. The tube regained its blood supply following detorsion. Prompt laparoscopy with detorsion should be aimed to preserve the affected fallopian tube and minimize impact on future fertility. In case of a paratubal cyst, the fallopian tube rotates on its own axis under the weight of the cyst [3]. Hence, paratubal cystectomy was performed following detorsion to prevent recurrence. Following detorsion, tubal length was found to be much longer than normal. Hence, salpingopexy was performed following paratubal cystectomy as another measure to prevent recurrence. Salpingopexy refers to the fixation of the tube by suturing to the broad ligament of the uterus or to the lateral pelvic wall [8]. Salpingopexy has been proposed to decrease the risk of tubal torsion recurrence, but there is limited evidence on its benefits or risks. Salpingopexy may cause shortening of the mesosalpinx resulting in reduced tubal mobility and reduction in blood supply to the adjacent ovary. It may affect the normal tubo-ovarian anatomy either by moving the adnexa outside the pelvis or by distorting the relationship between the ovary and the fimbriated portion of the tube [9]. However, on analyzing the risks versus benefits of salpingopexy, we went ahead with the procedure to minimize risk of recurrence of tubal torsion.

## Conclusions

IFTT is a rare cause of acute pain abdomen in adolescents and young females of reproductive age. Delay in diagnosis can lead to tubal necrosis and salpingectomy. Due to non-specific clinical and radiological findings, diagnosis is challenging; hence, prompt laparoscopy is needed in cases with suspicion. Timely intervention by laparoscopic detorsion can salvage the tube. Associated pathology should also be treated to prevent recurrence. Salpingopexy is a novel approach to prevent recurrence in cases particularly with long tubes.
